# Adolescent development of cortical oscillations: Power, phase, and support of cognitive maturation

**DOI:** 10.1371/journal.pbio.2004188

**Published:** 2018-11-30

**Authors:** Scott Marek, Brenden Tervo-Clemmens, Natalie Klein, William Foran, Avniel Singh Ghuman, Beatriz Luna

**Affiliations:** 1 Department of Neurology, Washington University in St. Louis, St. Louis, Missouri, United States of America; 2 Center for the Neural Basis of Cognition, Carnegie Mellon University, Pittsburgh, Pennsylvania, United States of America; 3 Department of Psychology, University of Pittsburgh, Pittsburgh, Pennsylvania, United States of America; 4 Department of Statistics, Carnegie Mellon University, Pittsburgh, Pennsylvania, United States of America; 5 Machine Learning Department, Carnegie Mellon University, Pittsburgh, Pennsylvania, United States of America; 6 Department of Psychiatry, University of Pittsburgh, Pittsburgh, Pennsylvania, United States of America; 7 Department of Neurological Surgery, University of Pittsburgh, Pittsburgh, Pennsylvania, United States of America; University of Oxford, United Kingdom of Great Britain and Northern Ireland

## Abstract

During adolescence, the integration of specialized functional brain networks related to cognitive control continues to increase. Slow frequency oscillations (4–10 Hz) have been shown to support cognitive control processes, especially within prefrontal regions. However, it is unclear how neural oscillations contribute to functional brain network development and improvements in cognitive control during adolescence. To bridge this gap, we employed magnetoencephalography (MEG) to explore changes in oscillatory power and phase coupling across cortical networks in a sample of 68 adolescents and young adults. We found a redistribution of power from lower to higher frequencies throughout adolescence, such that delta band (1–3 Hz) power decreased, whereas beta band power (14–16 and 22–26 Hz) increased. Delta band power decreased with age most strongly in association networks within the frontal lobe and operculum. Conversely, beta band power increased throughout development, most strongly in processing networks and the posterior cingulate cortex, a hub of the default mode (DM) network. In terms of phase, theta band (5–9 Hz) phase-locking robustly decreased with development, following an anterior-to-posterior gradient, with the greatest decoupling occurring between association networks. Additionally, decreased slow frequency phase-locking between frontolimbic regions was related to decreased impulsivity with age. Thus, greater decoupling of slow frequency oscillations may afford functional networks greater flexibility during the resting state to instantiate control when required.

## Author summary

During the transition from adolescence to adulthood, humans have decreases in impulsivity and increases in cognitive control. These behaviors are supported by a distributed set of brain regions, including the prefrontal cortex, that can be studied by with a variety of brain-imaging tools. Magnetoencephalography (MEG) is an approach that allows us to study spontaneous brain activity at the millisecond timescale, providing unique insight into local neural activity (power) and interactions between brain regions (estimated through phase-locking). Neural circuits exhibit oscillatory activity across a broad range of frequencies. Relatively slower-frequency (4–10 Hz) oscillations are thought to support cognitive control. We found that, during the transition from adolescence to adulthood, power was redistributed from slower frequencies to higher frequencies, with the greatest increase in faster frequency power in the posterior cingulate cortex. We also found that the phase-locking of prefrontal cortex theta band (5–9 Hz) oscillations decreases during adolescence. Mediation analysis of self-reported impulsive behavior suggests that band phase-locking contributes to decreases in impulsivity. This activity pattern may be an intrinsic marker for the ability for control-related brain regions to engage downstream processing networks. Our results indicate that spontaneous neural activity continues to be refined systematically during adolescence and contributes to cognitive maturation.

## Introduction

The transition from adolescence to adulthood is characterized by significant enhancements in brain function, supporting increased cognitive control and normative decreases in impulsivity [1,2]. Developmental task-based functional magnetic resonance imaging (fMRI) studies indicate that core regions supporting cognitive control (e.g., anterior cingulate cortex [ACC] and anterior insula [aIns]) are engaged in adolescence during cognitive tasks, but their blood oxygen level–dependent (BOLD) signal activation [3,4] and connectivity with other brain regions continue to increase into adulthood [5–7]. As such, brain networks supporting cognitive control are present prior to adolescence; however, the successful instantiation of cognitive control continues to improve [8]. Developmental resting-state fMRI (rs-fMRI) studies analyzing whole-brain connectivity patterns parallel this principle, such that the organization of functional brain networks is relatively stable by childhood [7,9,10], while integration (between-network functional connectivity) continues to refine well into late adolescence and early adulthood, supporting improvements in cognitive control [7].

The majority of developmental research on resting-state functional networks has utilized fMRI (see [11] for a review), providing the field a window into the development of resting-state networks at infra-slow frequencies (0.01–0.10 Hz). However, much less is known about the development of these networks at faster frequencies (i.e., 1–10 Hz oscillations) known to support the cognitive constructs that demonstrate a protracted development [12]. Because fMRI is not sensitive to this timescale of oscillation, magnetoencephalography (MEG) serves as a complementary tool to understand resting-state network development by allowing us to explore this relatively faster oscillatory range.

The correlation between electrophysiology and BOLD has been studied in both human and nonhuman primates, with a consistent finding of correlations between modalities in broadband gamma activity (40–100 Hz) within local neuronal pools during tasks [13,14]. Oscillations in this frequency range play a role in enabling local neuronal synchronization, whereas slower frequency (4–14 Hz) oscillations have been shown to support long-distance integration [15,16]. For example, synchronization of slow frequency oscillations within the frontoparietal (FP) network [17] are associated with cognitive control and have been shown to improve behavioral performance on control tasks [18,19]. Additionally, theta band activity (4–10 Hz) intensifies when control demands are increased [20]. Hence, slow frequency oscillations across control regions may contribute to top-down modulation of processing networks [12,21,22]. For example, long-range interactions from frontal to visual association regions during working memory retention and mental imagery evolved most strongly in the theta and alpha frequency range [23,24]. Moreover, evidence suggests that the prefrontal cortex leads the posterior parietal cortex in sustained visual attention tasks in the theta band [25]. Slower frequency oscillations, often in the theta band, have been shown to organize local neural activity in the gamma band, such that neurons tend to have greater firing rates in the trough of an ongoing slow frequency oscillation, providing a temporal template for neuronal communication [22,26]. As such, the phase of slower frequency oscillations, especially within the theta band, may be critical for coordination of neural activity over long distances [22,27].

In addition to task states, the electrophysiological correlates of control networks defined by BOLD fMRI during the resting state are becoming clearer. Resting-state BOLD networks correlate to the alpha and beta band, as measured with MEG [28]. There is additional evidence suggesting that correlations with BOLD may be greater at even slower frequencies, such as delta and theta bands (1–10 Hz) [29]. Recently, Hacker and colleagues characterized the spatial correspondence in humans of resting-state BOLD fMRI and band-limited power using electrocorticographic recordings, discovering frequency-specific oscillations within association networks in the slow frequency range (3–14 Hz) [30]. In sum, association networks map onto slower frequency oscillations (4–14 Hz) that may support coordinating activity of other brain networks.

Electrophysiological (i.e., electroencephalography [EEG]/MEG) studies have begun to offer insight into development changes in cortical oscillations. The majority of research concerning electrophysiological maturation across development has used EEG, finding age-related decreases in total power (total amount of activity across broadband frequencies) [31] and absolute power in each frequency band [31–34]. Additional work has shown that there is a redistribution of relative power (power in a given band in relation to total power across all frequencies) from lower to higher frequency bands [35], with frontal regions reaching adult levels of power after more posterior processing regions [31,32,36]. Similar posterior-to-anterior gradients have been observed using EEG measures of coherence, an index of regional coupling including both phase and amplitude components [37]. Notably, the curvilinear decreases in the delta and theta bands (i.e., 0.5–7 Hz) are highly correlated with gray matter volume decreases during adolescence [38]. Using MEG, increased amplitude correlations have been observed both within and between functional brain networks at rest throughout adolescence [39]. Although these studies have begun highlighting developmental trajectories of neural oscillations, the poor spatial specificity of EEG and lack of brain/behavior relationships utilizing MEG/EEG have limited our understanding of the regional and functional network development of oscillations and their potential contribution to cognitive development.

We sought to bridge this gap in the understanding of adolescent development, linking the age-related changes in brain network oscillations to cognitive development. In a sample of 68 adolescents and young adults (aged 14–31 years), we employed MEG to explore intrinsic properties related to oscillatory developmental within and between cortical networks, with regard to both power and phase. Specifically, within frequency intervals related to interareal neural interactions (1–49 Hz) [40,41], we examined regional and network-level oscillatory power and functional coupling of well-defined brain networks using the phase-locking value (PLV), similar to recent approaches [42]. Unlike correlation or coherence measures, the PLV ignores the amplitude (power) relationship between 2 oscillators. This enhances the ability to analyze phase relationships between brain regions, which is known to support interareal communication between large neuronal pools [26]. Interareal phase relationships in the theta band increase across multiple components of cognitive control [12], including working memory [43], error commission [44], and conflict.

Similar to previous EEG studies, we found a redistribution of regional power from slower delta band oscillations to faster beta band oscillations, with greater decreases in delta band power anteriorly in the cortex and greater increases in beta band power posteriorly. In terms of phase, we demonstrate age-related decreases in phase-locking of slow frequency (5–9 Hz) oscillations during adolescence, which followed a robust anterior-to-posterior gradient, with the greatest age-related changes in midline frontal regions, an area known have protracted cognitive development throughout adolescence [1,3,7]. Using a priori network membership, we show that the greatest developmental slow frequency decoupling occurred in higher-order association networks, relative to processing networks. Finally, we demonstrate that decoupling of slow frequency oscillations between anterior prefrontal regions and the anterior temporal lobe is related to self-reported impulsivity, a developmentally sensitive measure of cognitive control known to decrease robustly throughout adolescence.

## Results

### Developmental differences in global cortical phase-locking and power

**Fig 1:**
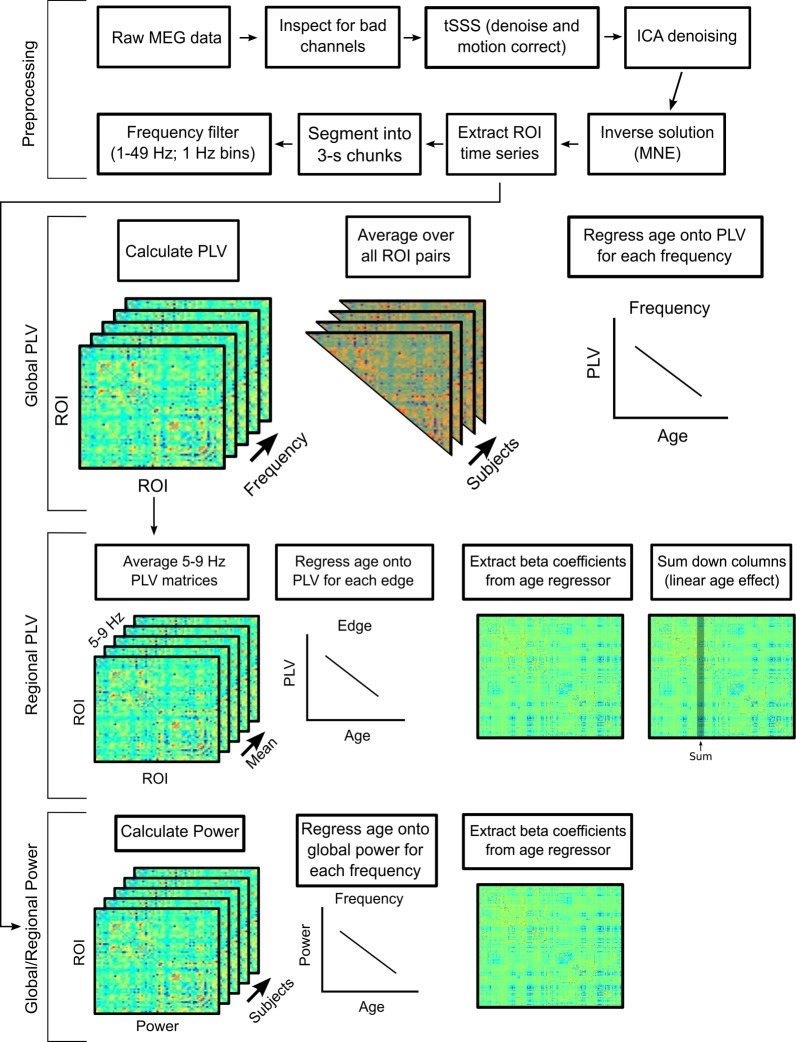
Workflow diagram. Preprocessing: Raw MEG and structural MRI data were preprocessed and coregistered. After surface ROI time series were extracted, a PLV was calculated for each frequency in the interval from 1–49 Hz, resulting in an ROI × ROI PLV matrix at each frequency interval for each subject. Global PLV: For each frequency, the mean PLV between all ROI pairs was calculated for each subject. Subject age was then regressed onto this global mean at each frequency to test for significant age effects, controlling for power. Regional PLV: Slow frequency (5–9 Hz) PLV matrices were averaged for each subject. Age was then regressed onto PLV for each edge of the matrix. The beta weight associated with age for every edge was extracted from each regression model. To summarize regional changes, we summed down the columns of the matrix, resulting in a composite linear age effect for each ROI. Global/regional power: Similar to the PLV pipeline, we calculated relative power for each ROI at each frequency interval. To obtain a global measure of power, we averaged power across all ROIs within a frequency band. For each region, we regressed power at a given frequency interval onto age and extracted the beta weight from the age regressor for additional analyses. Regional power estimates were examined for age effects and also included as nuisance regressors in all PLV × Age models. ICA, independent components analysis; MEG, magnetoencephalography; MNE, minimum-norm estimate; PLV, phase-locking value; ROI, region of interest; tSSS, temporal signal space separation.

In order to probe developmental changes in functional brain regions and networks, we used a previously defined functional parcellation established from rs-fMRI [45] to parcellate the cortical surface into 333 regions of interest (ROIs) in a sample of 68 individuals aged 14 to 31 years. For each ROI at each frequency (1–49 Hz; 1 Hz intervals), we calculated relative power to probe regional age-related changes in regional power and a PLV between each ROI pair to determine the age-related differences in degree of coupling between the phases of the oscillations between regions (see Fig 1 for workflow overview).

**Fig 2:**
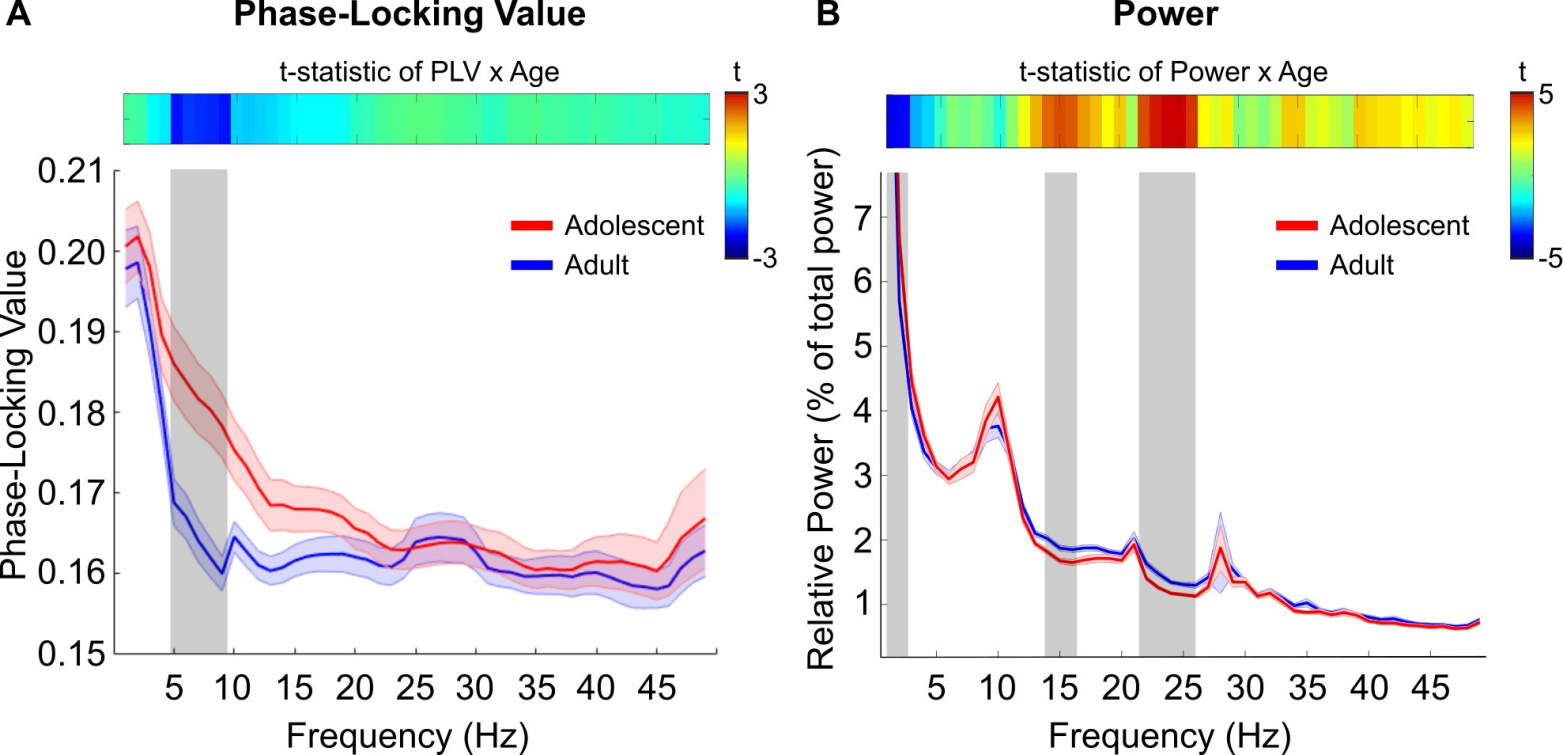
Developmental differences in global cortical phase-locking and power. (A) Across most frequency bands, adolescents displayed similar resting-state phase-locking to adults. However, in the 5–9 Hz frequency band, there was a significant linear decrease in phase-locking throughout development (gray shaded region; *p* < 0.05, FDR corrected). Top color bar represents the magnitude of the *t* statistic from the PLV × Age regression model. Data displayed categorically after segregation into 2 groups via a median split. (B) Power as a function of frequency. Delta band power significantly decreased with age, whereas beta band power significantly increased with age. Top color bar represents the magnitude of the *t* statistic from the Power × Age regression model. Data displayed categorically after segregation into 2 groups via a median split. In both (A) and (B), shaded gray patches represent frequency intervals demonstrating a significant linear relationship with age. Red and blue lines and shaded bars in the line plots represent the mean (solid line) and standard error of the mean (shaded patch around mean), respectively, in adolescents (red) and adults (blue). See S1 Data for individual data points. FDR, false discovery rate; PLV, phase-locking value.

First, we averaged the PLV matrices at each frequency across both ROI dimensions for each frequency and subject. This resulted in one global cortical PLV for each frequency, for each subject. There was no significant main effect of age predicting PLV (*β* = −0.0004, *t* = −1.255, *χ*^2^(1) = 1.576, *p* = 0.209). However, there was a significant age by frequency interaction predicting PLV (*χ*^2^[48] = 125.56, *p* < 0.001). A significant negative relationship between global PLV and age at each frequency interval between 5 and 9 Hz (all *p* < 0.05, false discovery rate [FDR] corrected) emerged, suggesting that phase relationships between regions in the 5–9 Hz frequency band become less coupled throughout adolescence (Fig 2A). No other frequency intervals showed a significant age-related change in PLV (all *p* > 0.05).

Similar to the PLV analysis, for each subject, we computed relative power at each frequency (1–49 Hz in 1 Hz intervals) for each ROI (see Methods for details). Similar to the PLV analysis, we obtained a measure of global power by averaging relative power across each ROI for each frequency. We observed a significant negative relationship between delta band power (1–3 Hz) and age (all *p* < 0.05, FDR corrected), such that delta band power decreased with age (Fig 2B). Conversely, beta band power (14–16 Hz and 22–26 Hz) significantly increased with age (all *p* < 0.05, FDR corrected), supporting previous developmental EEG studies noting a shift in power distribution, such that slower wave oscillations tend to shift towards relatively higher frequencies at rest [31,32,36]. There was no evidence for a significant relationship between 5–9 Hz power and age (*t* = −0.36, *p* = 0.71). Moreover, we did not observe a significant relationship between PLV and power (*t* = −0.01, *p* = 0.99). These results further support the notion that phase and power are largely orthogonal, providing complementary information in regard to the development of neural oscillations.

### Regional changes in PLV and power

To determine the anatomical locus of PLV decreases with age in the 5–9 Hz band, we averaged each individual subject’s PLV matrices in the 5–9 Hz frequency interval. Next, we regressed age onto each ROI pair’s PLV, controlling for motion and power (see Methods) and extracted the beta weight for age from each model. This resulted in a pairwise matrix of beta weights (beta matrix), representing the rate of change across development in slow frequency PLV for each ROI pair.

**Fig 3:**
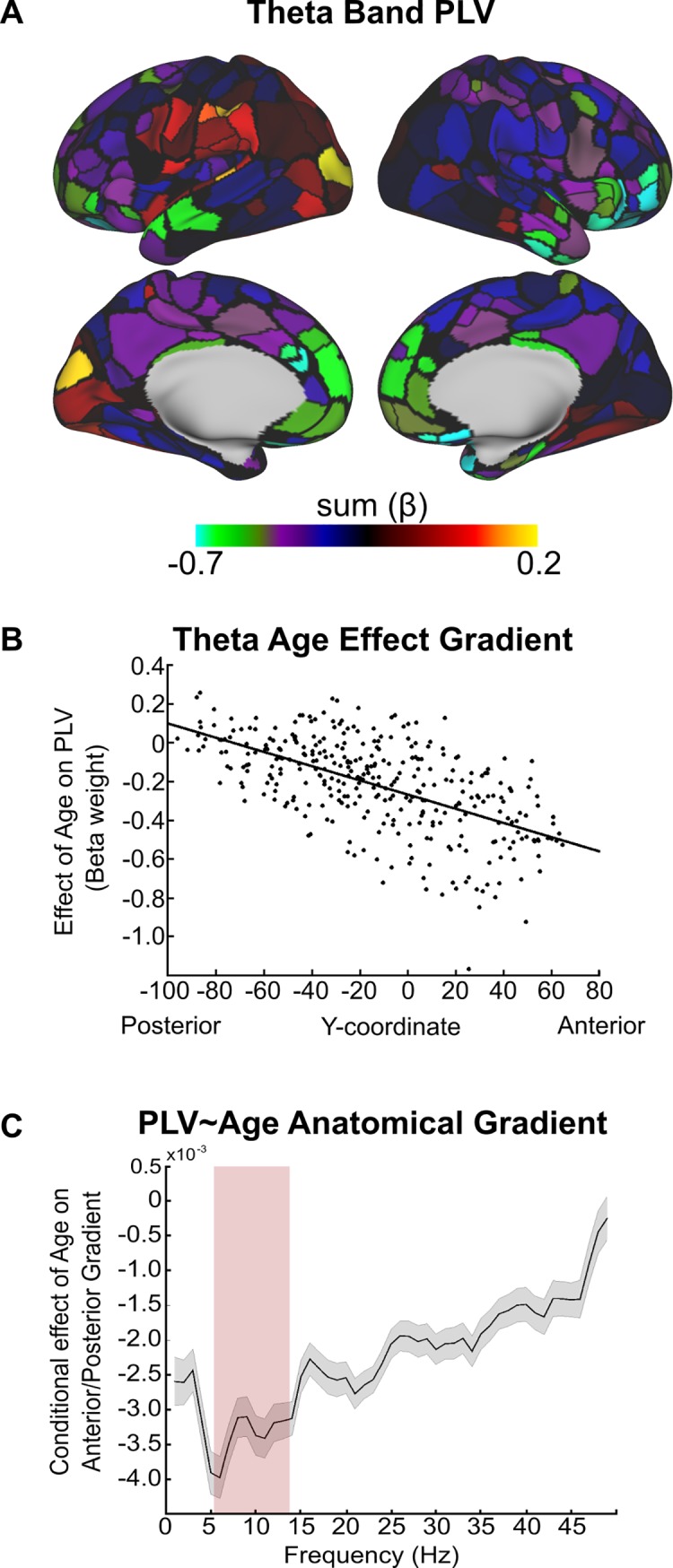
Regional age-related differences in phase-locking. (A) Regional age-related decreases in theta band phase-locking. (B) Scatter plot containing summed regional age effect (beta weight from theta PLV × Age model) as a function of the region’s anatomical y-coordinate center of mass. (C) PLV × Age anatomical gradient as a function of frequency. We found that the greatest anterior-to-posterior gradient developmental effect was in the 6–15 Hz regime. Gray error bars represent standard error of the model fit. Red shaded bar denotes theta/alpha regime. y-Axis represents the beta weight (slope) of the relationship between PLV and age with the anatomical y-coordinate of the region’s center of mass. See S1 Data for individual data points. PLV, phase-locking value.

We examined whether age-related changes in PLV demonstrated anatomical gradients across the cortex. To that end, we obtained a summary rate of change for each ROI by summing down the columns of the beta matrix and regressing each ROIs summed beta weight against its y-coordinate (in Montreal Neurological Institute [MNI] coordinate space) in each hemisphere and x-coordinate, separately. Average distance from each ROI to every other ROI and ROI surface area were included as nuisance regressors in all regression models to control for distance-dependent artifacts (i.e., anatomically proximal regions have artificially inflated PLV). Along the anterior-to-posterior axis, we observed a significant negative relationship between the summed beta weights and the y-coordinate (*t* = −13.19, *p* < 10^−10^), indicating a strong anterior-to-posterior gradient of PLV change, such that frontal regions showed greater decreases in theta band PLV (i.e., more decoupling) with age than posterior regions (Fig 3A and 3B). Regions undergoing the greatest decrease in PLV (top 5%) over development are rank ordered in Table 1. In the lateral-to-medial gradient, we observed a significant negative relationship between the summed beta weights and the x-coordinate in the left hemisphere (*t* = −6.97, *p* < 10^−10^) but only a trend in the right hemisphere (*t* = 2.01 *p* = 0.05), indicating slow frequency PLV decreased more rapidly with age along the medial wall. In sum, the greatest rate of decrease in slow frequency PLV occurred in midline frontal regions.

**Table 1:** Regions displaying the greatest rate of decrease in slow frequency phase-locking with age.

X	Y	Z	Hemi	Label	Network
−22.87	30.04	−17.67	L	Middle frontal gyrus	Default
35.67	36.83	−11.64	R	Middle frontal gyrus	Default
31.88	14.36	−30.62	R	Superior temporal gyrus	Default
22.60	31.59	−18.07	R	Middle frontal gyrus	Default
3.92	20.38	−21.68	R	Orbitofrontal gyrus	Default
2.74	38.45	−18.07	R	Orbitofrontal gyrus	Default
−11.93	24.61	−18.61	L	Medial frontal gyrus	Default
37.93	6.63	−39.65	R	Middle temporal gyrus	Default
41.73	49.58	−7.32	R	Middle frontal gyrus	FP
45.60	28.86	−7.42	R	Inferior frontal gyrus	FP
39.61	47.59	8.39	R	Middle frontal gyrus	FP
−7.24	33.40	23.28	L	ACC	FP
30.20	18.99	−16.89	R	Inferior frontal gyrus	Ventral attention
12.40	25.56	−24.03	R	Orbitofrontal gyrus	None
25.06	7.74	−16.41	R	Subcallosal gyrus	None
51.90	−10.20	−35.81	R	Inferior temporal gyrus	None

**Abbreviations:** ACC, anterior cingulate cortex; FP, Frontoparietal; Hemi, hemisphere.

**Fig 4:**
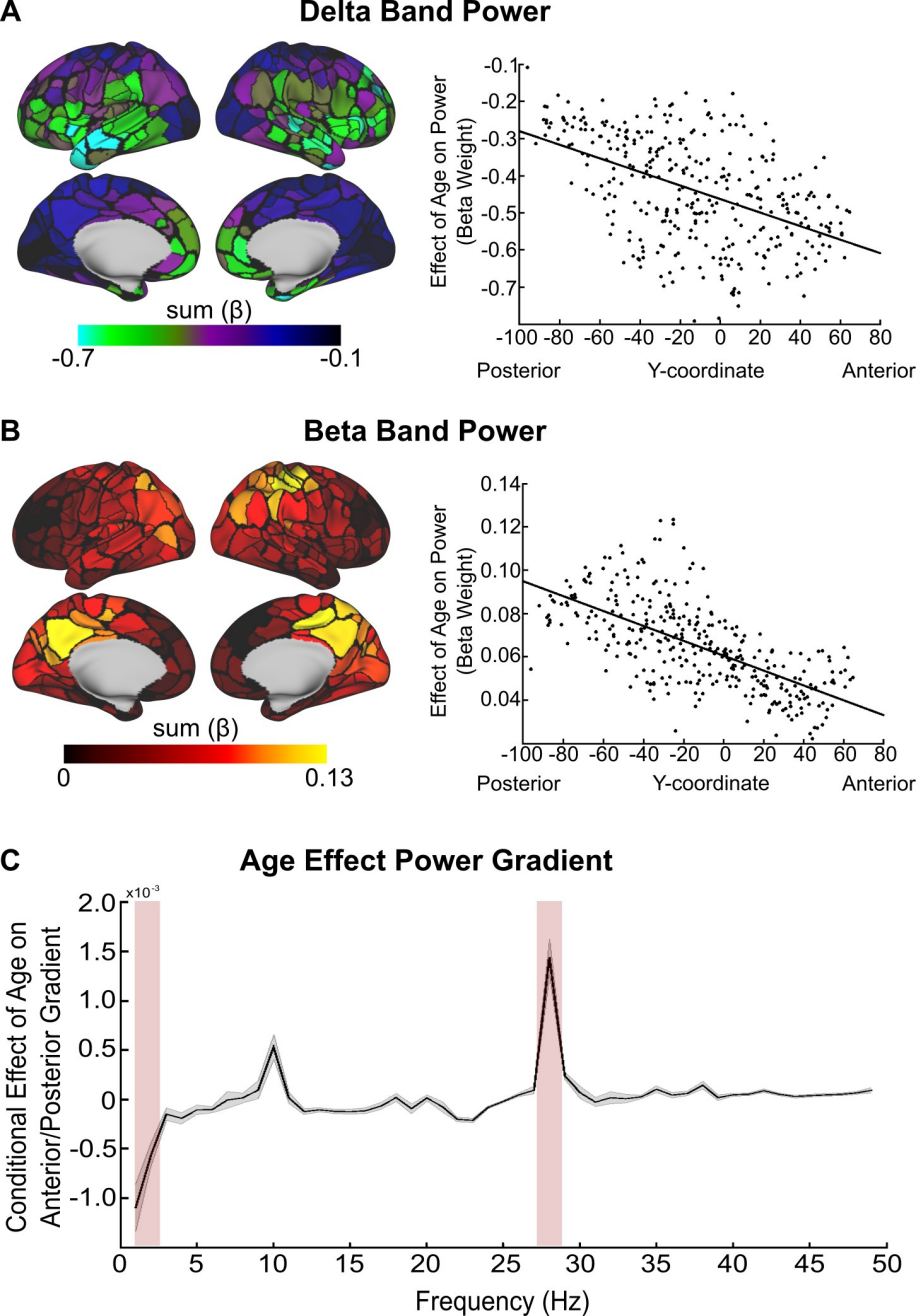
Regional age-related differences in power. (A) Regional age-related decreases in delta band power. (B) Regional age-related increases in beta band power. Scatter plots contain summed regional age effect (beta weight from Power × Age model) as a function of the region’s anatomical y-coordinate center of mass. (C) Power × Age anatomical gradient as a function of frequency. Error bars represent standard error of the model fit. Red shaded bar denotes delta and beta band regimes from panels A and B. y-Axis represents the beta weight (slope) of the relationship between power and age with the anatomical y-coordinate of the region’s center of mass. See S1 Data for individual data points.

In addition to the PLV analysis, we also characterized regional changes in power throughout adolescence. For each region, we summed the beta weights across frequencies demonstrating a significant Power × Age relationship in Fig 2B, for delta and beta bands separately. Similar to slow frequency PLV, delta band power demonstrated a significant anterior-to-posterior gradient (*t* = −10.33, *p* < 0.0001), with the largest age-related decreases in delta power occurring in frontal regions, especially in the frontal operculum (Fig 4A). In contrast to delta power, developmental beta band increases in power followed a posterior-to-anterior gradient (*t* = 15.86, *p* < 0.0001), such that the greatest developmental increases in beta band power occurred in medial and lateral parietal regions (Fig 4B). Of note, the posterior cingulate cortex, a hub of the default mode (DM) network, demonstrated the greatest age-related increase in beta band power. Power in the 5–9 Hz frequency interval did not demonstrate any significant age-related increases or decreases (*t* = −0.36, *p* = 0.71), nor did 5–9 Hz power demonstrate any significant developmental anterior-to-posterior gradients (*t* = −1.70, *p* = 0.09).

### Age-related changes in PLV and power are frequency specific and specific to the resting state

To assess developmental changes in the anterior-to-posterior gradient of PLV in other frequency bands, for each subject and each ROI, we regressed age onto PLV and extracted the resulting beta weight for age. Beta weight matrices were generated for each frequency interval (see Methods), summed, and regressed against the ROI’s y-coordinate. We then extracted the beta weight from the y-coordinate regressor in each regression model and plotted this as a function of frequency (Fig 3C). Slow frequency age-related decreases in PLV were most prominent at 6 Hz. To quantify these results statistically, we tested for significant differences in the correlation between ROI beta weights and anterior-to-posterior gradients between a given frequency interval (in 5 Hz bins) by comparing the slopes (i.e., beta weights) of the regression models from each frequency interval to the 6–10 Hz interval (see Methods for more details). A significant difference would be reflected in a z-statistic > 1.645, *p* < 0.05, one-tailed, indicating that the 6–10 band had a significantly greater negative slope between the summed beta weights for PLV × Age and the anatomical y-coordinate of the region. We did not find evidence for a significant difference for the alpha range (intervals from 11–15 Hz; *z* = 0.13, *p* > 0.05). However, for frequencies less than 6 Hz and greater than 15 Hz, we did find a significant interaction (all *z* > 1.645, *p* < 0.05), indicating that the greatest gradients in PLV occur within the theta and alpha band regime.

To quantify developmental changes in the anterior-to-posterior gradient of power across all frequency bands, for each subject and each ROI, we regressed age onto power and extracted the resulting beta weight for age. As in the PLV analysis, beta weight matrices were generated for each frequency interval (see Methods) and were regressed against the ROI’s y-coordinate. We observed a negative gradient in the delta regime, whereas a positive gradient existed in the beta band (Fig 4C). Thus, age-related decreases in delta band power were most prominent in frontal regions, whereas age-related changes in beta band power were most prominent in posterior regions.

Next, we aimed to determine whether our developmental effect of an anterior-to-posterior gradients of PLV and power differences with development were specific to the resting state versus a task state. To this end, we analyzed data from the maintenance period of a working memory paradigm in a subset of our sample (*n* = 28; details of MEG task methods and results in Methods). After extracting pairwise PLVs and regional power for each subject and frequency band within the 5–9 Hz band, we averaged across frequency bands, resulting in 1 phase-locking matrix per subject. Paralleling the resting-state analysis, we regressed age on each pairwise PLV across subjects, controlling for subject head motion. We extracted the beta weight from the age regressor, resulting in a beta weight matrix, representing linear effects of age on changes in PLV during working memory maintenance. To test for an anterior-to-posterior effect as was observed during the resting state, we summed down the columns and regressed the ROI’s y-coordinate on this summed linear age effect. We did not observe an anterior-to-posterior gradient during working memory maintenance (*t* = −0.02, *p* = 0.98). Moreover, we did observe the anterior-to-posterior gradient in this subset of subjects (*t* = −9.31, *p* < 10^−10^) during rest. These findings suggest that the strong decreases in 5–9 Hz phase-locking in frontal regions likely are specific to the resting state. Similar to PLV, the age-related effects in delta and beta power were specific to the resting state. We calculated power during the maintenance period of the working memory task across the delta (1–3 Hz) and beta band (14–16 Hz and 22–26 Hz). For each frequency interval and each ROI, we regressed age against power and extracted the beta weights from the age regressor. For each frequency interval, we regressed the y-coordinate against the beta weights. We did not observe an anatomical gradient within the delta band or beta band during the maintenance period of the task (all *p* > 0.05, FDR corrected), suggesting that age-related effects in power are also specific to the resting state. Together, these results indicate that adolescence is characterized by frequency-specific changes in PLV and power that are specific to the resting state.

### Network-level changes in PLV and power

**Fig 5:**
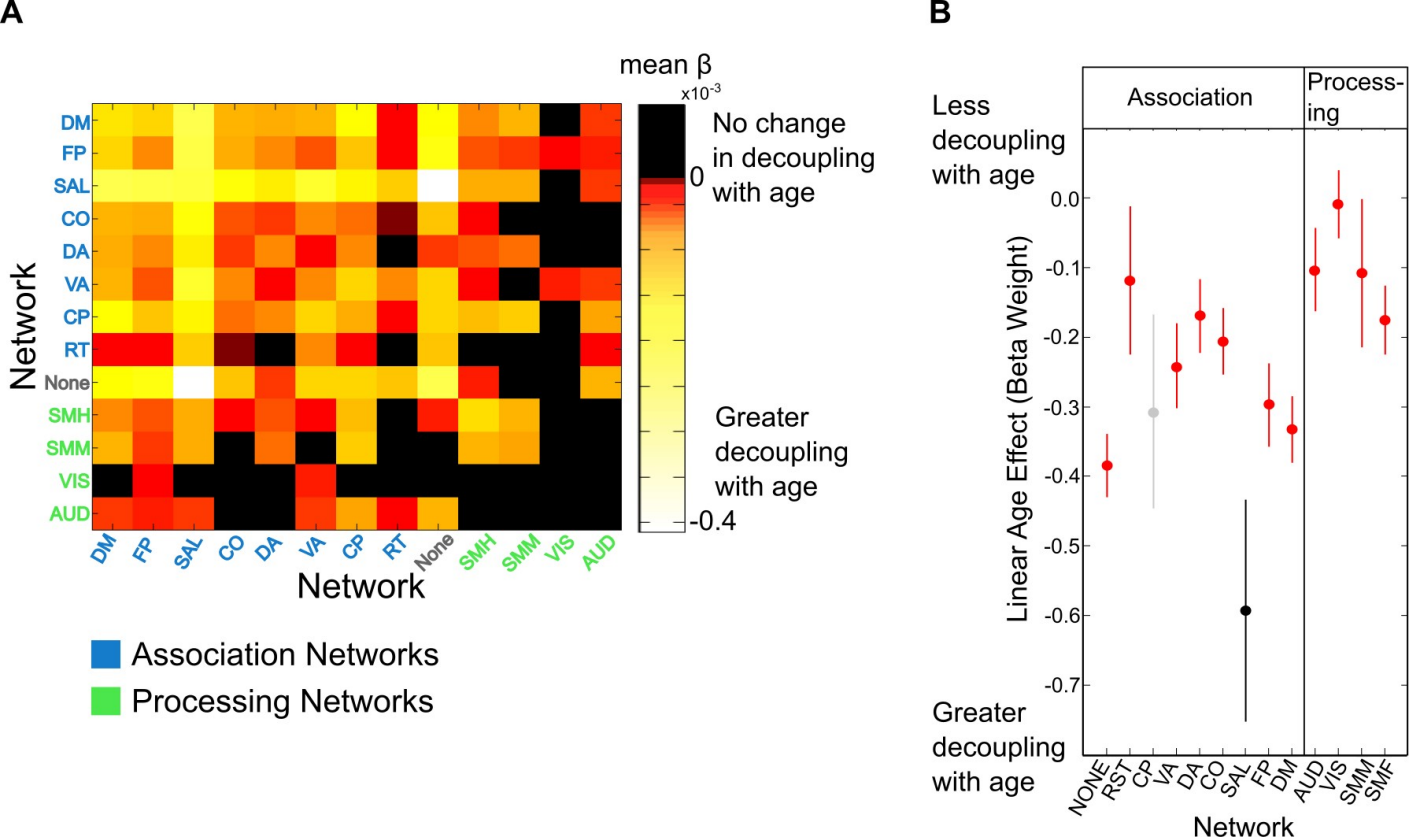
Network changes in phase-locking. (A) Age-related decreases in phase-locking tended to be within and between association networks (e.g., DM, FP, and SAL), while within- and between-network oscillations involving processing networks remained relatively stable. (B) Age-related increases in slow frequency decoupling were greater in association networks than in processing networks (*p* = 10^−9^). Oscillations in the SAL network became significantly more decoupled compared to any other association or processing network, with the exception of the CP network (all *p* < 0.05, corrected). See S1 Data for individual data points. AUD, Auditory; CO, Cinguloopercular; CP, Cinguloparietal; DA, Dorsal Attention; DM, Default Mode; FP, Frontoparietal; NONE, Unknown; RT, Retrospenial Temporal; SAL, Salience; SMH, Somatomotor Hand; SMM, Somatomotor Mouth; VA, Ventral Attention; VIS, Visual.

In addition to specific regional changes in PLV, we aimed to characterize developmental changes in PLV as a function of networks [45]. For each network combination (e.g., DM-DM, DM-FP, etc.), we obtained the mean beta weight of the linear effect of age on PLV for all ROI pairs of the networks being compared. The resulting heat map is shown in Fig 5A. We then performed a one-way ANOVA to quantitatively assess whether some networks experienced a greater rate of change in PLV with age compared to others. Here, we submitted summed beta weights of within-network interactions (e.g., DM to DM) to the ANOVA. As determined by the ANOVA test, there was a significant difference in the summed beta weight for age effects at the network level (*F*_[12,320]_ = 9.57, *p* = 10^−10^). A subsequent post hoc analysis revealed that the negative linear age effect was greatest for the salience (SAL) network compared to any other network (*p* < 0.05) (Fig 5B). More generally, a *t* test between the beta weights within association networks and the beta weights within processing networks revealed that PLV within association networks decreased with age significantly more compared to processing networks (*t* = −6.51, *p* < 0.001).

To make inferences concerning significant developmental differences in delta band and beta band power at the network level, we performed a one-way ANOVA on the beta weights by grouping the regions according to a priori network affiliation for the delta and beta regime, separately. With respect to the delta band, we found a significant difference in the average beta weight for age effects at the network level (*F*_[12,320]_ = 22.71, *p* = 10^−36^). A subsequent post hoc analysis revealed that age-related decreases in delta power within networks were greatest for the auditory, SAL, cinguloopercular, and FP networks (all post hoc comparisons were corrected for multiple comparisons using the Tukey method). For complete post hoc results, see Table 2.

**Table 2:** Comparisons between functional networks in magnitude of age-related change in delta band power.

	None	RST	CP	VA	DA	CO	AUD	VIS	SMM	SMH	SAL	FP	DM
**DM**	0.053	−0.124	−.165	0.025	−0.075	0.037	0.058	−0.199	−0.083	−0.178	0.063	0.021	**−0.477**
**FP**	0.032	−0.145	−.186	0.004	−0.096	0.016	0.037	−0.220	−0.104	−0.199	0.042	**−0.498**
**SAL**	−0.010	−0.187	−.228	−0.038	−0.139	−0.026	−0.005	−0.262	−0.146	−0.241	**−0.540**
**SMH**	0.231	0.054	.013	0.203	0.103	0.215	0.236	−0.021	0.095	**−0.299**
**SMM**	0.136	−0.041	−.082	0.108	0.008	0.120	0.141	−0.116	**−0.394**
**VIS**	0.252	0.075	.034	0.224	0.124	0.236	0.258	**−0.278**
**AUD**	−0.005	−0.182	−.223	−0.033	−0.134	−0.021	**−0.536**
**CO**	0.016	−0.161	−.202	−0.012	−0.113	**−0.515**
**DA**	0.128	−0.049	−.089	0.101	**−0.402**
**VA**	0.028	−0.149	−.190	**−0.503**
**CP**	0.218	0.041	**−.313**
**RST**	0.178	**−0.353**
**None**	**−0.531**

Each cell represents the network difference between the mean of the summed beta weights from the Delta power × Age model. Cells highlighted in blue indicate a significant difference with development. The direction of the difference is Row-Column. Diagonal elements contain the mean beta weight (linear age effect of Power × Age) for each network. First row and column refer to the functional networks.

**Abbreviations:** AUD, Auditory; CO, Cinguloopercular; CP, Cinguloparietal; DA, Dorsal Attention; DM, Default Mode; FP, Frontoparietal; None, Unknown; RST, Retrosplenial Temporal; SAL, Salience; SMH, Somatomotor Hand; SMM, Somatomotor Mouth; VA, Ventral Attention; VIS, Visual.

With respect to beta band power, we also found a significant difference in the average beta weight for age-related differences at the network level (*F*_[12,320]_ = 12.52, *p* = 10^−20^). A subsequent post hoc analysis revealed that age-related increases in beta power were greatest for somatomotor, auditory, and visual networks. For complete post hoc results, see Table 3.

**Table 3:** Comparisons between functional networks in magnitude of age-related change in beta band power.

	None	RST	CP	VA	DA	CO	AUD	VIS	SMM	SMH	SAL	FP	DM
**DM**	0.010	−0.003	−0.032	0.003	−0.011	−0.003	−0.014	−0.017	−0.017	−0.024	0.015	0.001	**0.058**
**FP**	0.010	−0.004	−0.032	0.003	−0.011	−0.003	−0.014	−0.017	−0.017	−0.024	0.015	**0.059**
**SAL**	−0.005	−0.019	−0.048	−0.013	−0.026	−0.018	−0.030	−0.032	−0.032	−0.040	**0.043**
**SMH**	0.035	0.021	−0.008	0.027	0.013	0.021	0.010	0.008	0.007	**0.083**
**SMM**	0.027	0.013	−0.016	0.020	0.006	0.014	0.003	0.001	**0.076**
**VIS**	0.027	0.013	−0.016	0.019	0.005	0.014	0.002	**0.075**
**AUD**	0.025	0.011	−0.018	0.017	0.003	0.011	**0.073**
**CO**	0.013	−0.001	−0.030	0.005	−0.008	**0.062**
**DA**	0.021	0.008	−0.021	0.014	**0.069**
**VA**	0.008	−0.006	−0.035	**0.056**
**CP**	0.043	0.029	**0.091**
**RST**	0.014	**0.062**
**None**	**0.048**

Each cell represents the network difference between the mean of the summed beta weights from the Beta power × Age model. Cells highlighted in blue indicate a significant difference with development. The direction of the difference is Row-Column. Diagonal elements contain the mean beta weight (linear age effect of Power × Age) for each network. First row and column refer to the functional networks.

**Abbreviations:** AUD, Auditory; CO, Cinguloopercular; CP, Cinguloparietal; DA, Dorsal Attention; DM, Default Mode; FP, Frontoparietal; None, Unknown; RST, Retrosplenial Temporal; SAL, Salience; SMH, Somatomotor Hand; SMM, Somatomotor Mouth; VA, Ventral Attention; VIS, Visual.

### Pairwise decreases in resting-state phase-locking

**Fig 6:**
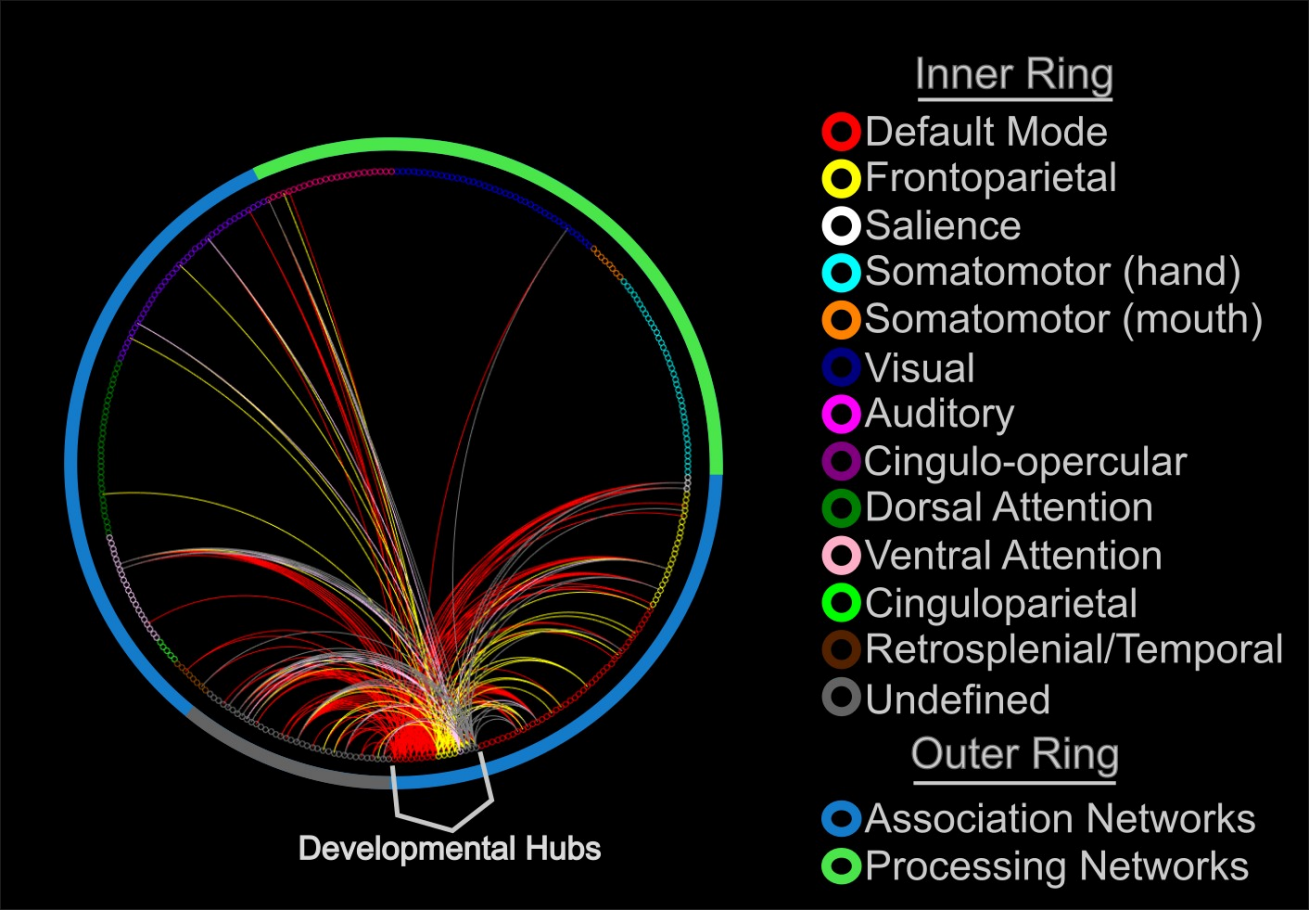
Pairwise age-related decreases in resting-state phase-locking. Pairwise increases in decoupling between the top 5% of brain regions that showed age-related increases in decoupling (developmental hubs) and their respective top 5% pairwise interactions. Regions (little circles) are colored by the network to which they are affiliated. Link color represents the network affiliation to which the developmental hub belonged. The most significant pairwise increases occurred between regions of the DM, and FP networks to other association networks. DM, Default Mode; FP, Frontoparietal.

After determining the gradient and locus of decreased phase coupling from adolescence to adulthood, we analyzed specific ROI pairs driving this decrease. Specifically, we aimed to determine the specific pairwise interactions that contributed to the greatest rate of 5–9 Hz oscillatory decoupling. We first identified the top 5% of ROIs that showed the greatest rate of 5–9 Hz decoupling (developmental hubs) from the regional analysis. From those ROIs, we extracted the top 5% of negative beta weights and plotted the connections, with ROIs grouped by networks (Fig 6), as assigned by [45]. All ROIs from the regional analysis were within higher-order association networks, with 8 belonging to the DM network, 3 belonging to the FP network, 1 belonging to the SAL network, 1 belonging to the ventral attention (VA) network, and 3 belonging to an undefined network, though all regions were within anterior portions of the frontal lobe and are considered part of the limbic network in other parcellations (e.g., ref [46]). With the exception of 2 links, all links from these developmental hubs were to regions of other association networks, indicating that pairwise decreases in 5–9 Hz coupling are largely specific to association networks.

### PLV mediation of age and impulsivity

We have demonstrated a strong decrease in 5–9 Hz PLV within midline frontal regions. Given the role of anterior prefrontal cortex and anterior temporal lobes in impulse control [47] and the role of theta (4–10 Hz) oscillations in cognitive control [12], we sought to determine whether decreases in frontal slow frequency PLV were related to decreased impulsivity throughout adolescence. The UPPS-P Impulsive Behavior Scale is a validated self-report 59-item measure of impulsivity [48]. Items are endorsed on a 4-point scale from 1 (agree strongly) to 4 (disagree strongly). After appropriate reverse scoring, scores for each item range from 1 (non-impulsive answer) to 4 (high level of self-reported impulsivity). The UPPS-P can provide scores from specific subscales (e.g., Urgency, Lack of Premeditation, Lack of Perseverance, Sensation Seeking). In the current analysis, we utilized a total impulsivity measure (mean across all items) to increase the precision of each subject’s estimate. Within our sample, total impulsivity scores from the UPPS-P scale (M = 2.02, SD = 0.35; Range [1.32, 2.75]) were consistent with normative variability in impulsivity as reported in previous work [49]. Furthermore, the Cronbach α for the total impulsivity measure in our sample was 0.93, indicating excellent internal consistency. Total impulsivity was negatively associated with age (*β* = −0.333, *t* = −2.74, *p* = 0.008), such that impulsivity decreased significantly with development. To obtain a cluster of regions that significantly decreased in PLV as a function of age, we submitted the individual subject matrices to the network-based statistic (NBS) [50]. The NBS is a common tool used in rs-fMRI studies to identify clusters of suprathreshold links displaying a similar effect (e.g., increasing or decreasing PLV with age). It seeks to control family-wise error rate when mass univariate testing occurs, as in the case of running regression analyses on each ROI pair. Briefly, a test statistic is generated for each ROI pair’s PLV as a function of age. A cluster is identified using a breadth first search, followed by permutation testing to significance based on a cluster’s size.

**Fig 7:**
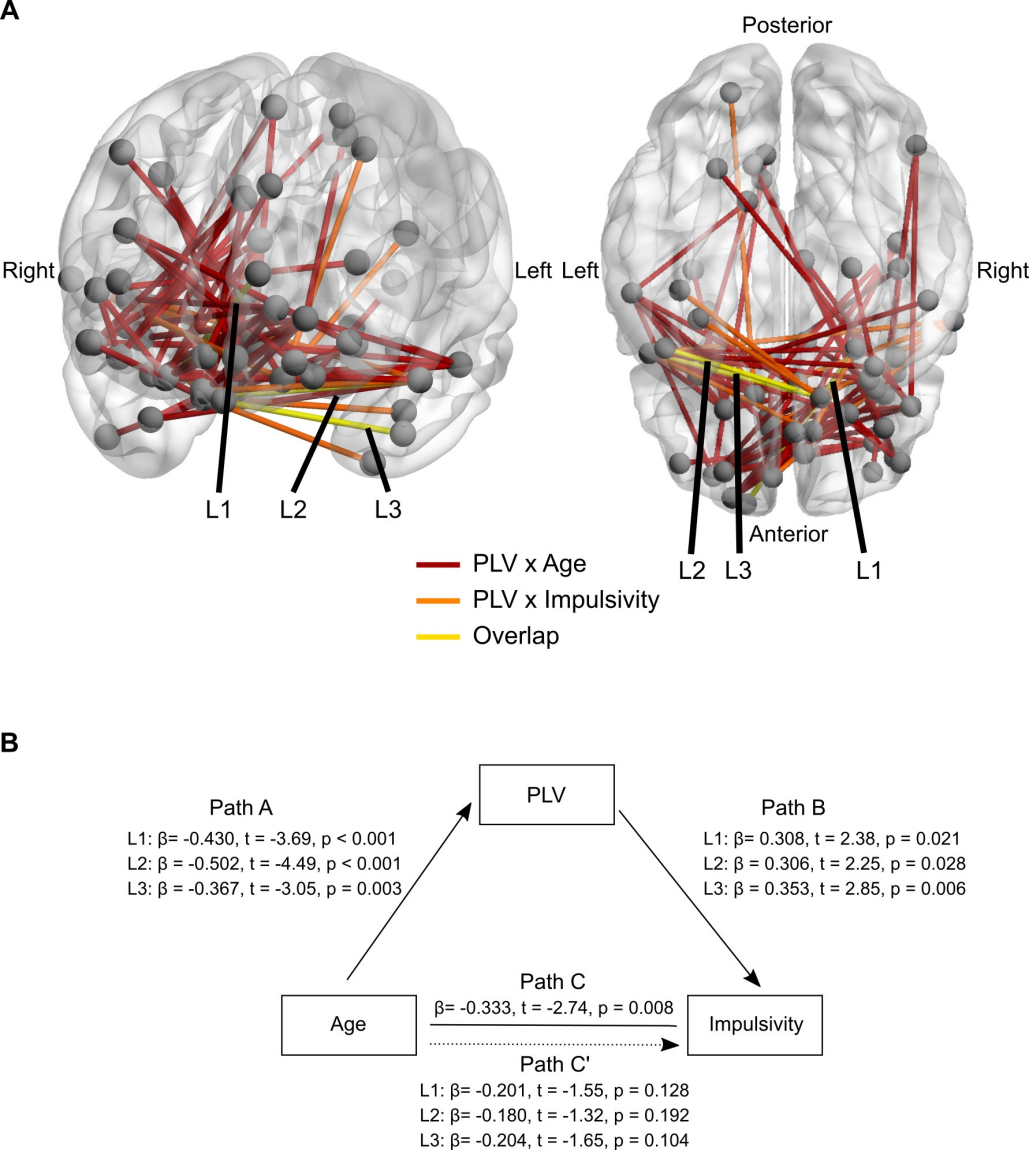
Frontolimbic 5–9 Hz phase-locking is related to decreased impulsivity during adolescence. (A) Anatomical location PLV × Age and PLV × Impulsivity relationships. Red links denote the significant PLV × Age NBS cluster. Orange links denote the significant PLV × Impulsivity NBS cluster. Yellow links denote overlap between the 2 clusters. These overlapping links were tested for mediation. (B) Mediation model including statistics for specific paths. Note PLV of these 3 interactions fully mediated the relationship between age and impulsivity (difference in *p*-values between path C and paths in C’), confirming overlap of clusters. NBS, network-based statistic; PLV, phase-locking value.

A cluster composed of 49 regions with 122 links survived the permutation test (1,000 resamples; red links in Fig 7A). Similarly, we performed a median split on impulsivity to break the sample into a high impulsivity group and a low impulsivity group. Individual subject matrices were once again submitted to the NBS, controlling for age. A cluster composed of 13 regions with 14 links survived the permutation test (1,000 resamples; orange links in Fig 7A). Three links comprising 5 distinct regions overlapped between the 2 clusters (PLV × Age and PLV × Impulsivity; yellow links in Fig 7A). For statistical confirmation of overlap between PLV and age with PLV and impulsivity, we subsequently submitted to 3 separate mediation analyses. The fist link (L_1_) was between the left superior frontal gyrus (MNI coordinates: −15.05, 64.73, 13.29) and the right inferior frontal gyrus (MNI coordinates: 25.07, 7.38, −16.41), the second link (L_2_) was between the left temporal gyrus (MNI coordinates: −50.60, 9.26, −18.71) and right medial frontal gyrus (MNI coordinates: 12.40, 25.55, −16.38), and the third link (L_3_) was between the left middle temporal gyrus (MNI coordinates: −44.87, 7.38, −34.85) and the right medial frontal gyrus (MNI coordinates: 12.40, 25.55, −16.38). As a separate means of dimensionality reduction more focused on the a priori network organization, as well as the strong 5–9 Hz decoupling within the SAL network, we also tested mean SAL network PLV as a mediator between age and impulsivity. Mean SAL network PLV was not associated with UPPS-P total impulsivity scores while co-varying age (*β* = −0.183, *t* = −1.45, *p* = 0.152).

In addition to PLV, we also tested delta band power and beta band power for meditation in the relationship between age and impulsivity. Neither delta (minimum *p* = 0.47, FDR corrected) nor beta-power (minimum *p* = 0.90, FDR corrected) in any node significantly mediated the relationship between age and impulsivity. Together, these results indicate that resting-state slow frequency phase-locking, not power, contributes to age-related decreases in impulsivity.

Mediation analysis on each link separately revealed that partialing out the variance of each of the 3 ROI pairs significantly attenuated the relationship between age and impulsivity (indirect pathway [path ab], L_1_: *β* = −0.133 [95% CI −0.244 to −0.017], *p* = 0.03; L_2_: β = −0.154 [95% CI to −0.322, −0.023], *p* = 0.02; L_3_: β = −0.130 [95% CI, −0.251 to −0.036], *p* = 0.003). For statistics on individual paths, see Fig 7B. These findings suggest the observed age-related decreases in impulsivity is, in part, accounted for by the decoupling of slow frequency oscillations during the resting state between the anterior prefrontal cortex and the anterior temporal lobe. However, care should be taken when interpreting the mediation effects, as links demonstrating significant mediation did not survive multiple comparisons corrected when all PLV × Age links were tested together. Regardless, overlapping links between brain/behavior and brain/age relationship suggest that slow frequency PLV, in part, contributes declining impulsivity during adolescence.

## Discussion

Interactions between functional brain networks demonstrate a protracted development well into adolescence and early adulthood [6,7,10] and have been shown to support the maturation of cognitive control [7]. However, the development of resting-state network oscillations and their contribution to cognitive development have not been explored. We found a decrease in theta band (5–9 Hz) phase coupling that was strongest in midline frontal regions, especially in association networks. In parallel, many of the strongest pairwise decrease in resting-state theta coupling occurred between regions affiliated with the DM, FP, and SAL networks. Furthermore, decreased slow frequency coupling between anterior frontal and temporal lobe regions was related to decreased impulsivity with development, providing an oscillatory contribution for decreased impulsivity throughout development.

In terms of oscillatory power, we found a redistribution of power from slower delta oscillations to faster beta oscillations. These findings support and extend prior resting-state EEG [31,34], and concurrent EEG-fMRI studies [51] have reported significant developmental decreases in delta power and increases in beta power [35]. Here, we extend these findings through source localization enabling characterizing of these developmental changes in terms or regions and functional networks. Specifically, there were significant age-related decreases in delta power, most strongly in frontal and opercular regions comprising the SAL and cinguloopercular networks. Conversely, there were significant age-related increases in beta power, most prominent in processing networks. The posterior cingulate cortex, a hub of the DM, demonstrated the greatest age-related increase in beta band power. The DM network demonstrates a protracted development in BOLD connectivity [52], supporting increased specialization and integration of this network with other functional networks [53].

A canonical feature of electrophysiological estimates of power and phase during the resting state is the dominance of oscillations in posterior regions of the brain. The negative slope of age-related decreases as a function of the posterior-to-anterior gradient suggests that frontal regions are becoming more decoupled broadly but most prominently, and statistically significantly, for the 5–9 Hz (theta) band. The post hoc analysis in which we tested for significant differences in the correlation between ROI beta weights and anterior-to-posterior gradients between a given frequency interval (in 5 Hz bins) statistically supports the notion that the anterior-to-posterior gradient is most prominent for the 5–15 Hz frequency interval, which includes the theta (5–9 Hz) interval in which we observed a significant negative relationship between PLV and age. Thus, the gradient analyses, in conjunction with Fig 3A, provide evidence that theta band (5–9 Hz) decoupling is most prominent in midline prefrontal regions.

Similar to early electrophysiological work using EEG to study coherence between cortical lobes [54], we found a protracted development of control networks within the 5–9 Hz frequency interval, particularly within the SAL network, comprised of the anterior cingulate and aIns. Both of these regions are anatomical and functional hubs of the cortex [55,56], with anatomical connections to several major brain networks. Generally, theta band oscillations have been shown to organize higher frequency activity, providing a temporal template for neuronal communication [22,26]. Thus, the phase of theta band oscillations may be critical for the coordination of neural activity [22,27]. Supporting this supposition, a large body of evidence suggests oscillations arising from the SAL network entrain disparate control networks when the need for control is realized [12]. Because adolescence is marked by substantial reductions in behavioral variability that is reliant on control networks [4,57,58], we propose that age-related frontal theta decoupling during the resting state may support the enhanced ability for adults to reliably instantiate control and coordinate regulatory control networks. In support of this, BOLD connectivity studies have found increases in the spatial variability of control and attention networks with development but stability of processing networks [53].

A cluster of frontolimbic regions in anterior prefrontal and anterior temporal lobes also displayed slow frequency decoupling with development. Interactions between these frontolimbic regions and the SAL network had the greatest rate of decoupling of any within- or between-network comparison (Fig 5A). Frontolimbic connectivity is often prescribed a role in impulse control, and when structurally lesioned, leads to greater impulsivity [59,60]. Additionally, recent diffusion tensor imaging and fMRI evidence suggests that frontolimbic connectivity decreases both structurally and functionally throughout adolescence [61,62]. Here, we showed evidence that several interactions between frontolimbic regions were related to impulsivity and also demonstrated significant slow frequency decoupling, confirmed by a mediation analysis. Theta band (5–9 Hz) oscillations may be the mechanism by which these regions communicate to execute impulse control given the role of theta oscillations in the instantiation of cognitive control [12]. Lending support to this proposal, theta band activity tends to flow from frontal regions to more posterior regions [63], suggesting a possible causal association.

Phase-locking should be largely unaffected by power within the same frequency band (but see ref [64]). While age-related changes in PLV and power are related to overarching processes of brain maturation through adolescence, they inform different levels of neural processing. While frequency changes reflect local circuit modifications, PLV reflects the possible interareal effects of these circuit modifications, specifically with regard to coupling across brain regions. Distinct circuit and systems-level modifications are evident through adolescence that would have direct effects on both frequency and coupling (see [65] for a review). At the circuit level, power may be directly affected by maturation inhibitory circuitry supported by increases in GABA, particularly parvalbumin interneurons within the prefrontal cortex [66–68], resulting in greater power within the beta/gamma frequency range [69]. In parallel, and likely indirectly related, there are systems-level changes in specialization of existing connections, such as age-related decreases in frontolimbic connectivity [10,61], that would contribute to the decoupling of slow wave oscillations affecting PLV. As such, developmental decreases in phase-locking may reflect stochastic resonance and/or neural flexibility [70]. If the brain were to maintain a rigid configuration of interactions at this timescale during rest, the ability to explore and switch between brain states would be undermined. Indeed, a prominent theory on the nature of resting state proposes that it serves to allow the sampling of multiple network configurations along an anatomical backbone [71,72]. If this is the case, functional brain networks require flexibility in the form of imperfectly coupled oscillators (i.e., variability) to maintain dynamics in networks at this timescale (millisecond). Several studies have found evidence for increased cortical variability throughout development [70,73,74]. Our findings here support these fMRI-based findings in that decreased phase-locking may represent an overall age-related increase in variability [40,75,76], as well as an overall increase in signal complexity.

A potential limitation of the current study is the depth sensitivity of MEG. The signal-to-noise ratio (SNR) falls with increasing distance from the MEG sensors. However, this limitation exists across all subjects, and thus all ages considered in this study. Given this limitation, we were able to demonstrate decreases in theta band phase-locking within medial wall structures that showed specificity to the resting state versus a working memory task-state.

In sum, our results support and extend previous electrophysiological work characterizing the development of oscillatory power, such that power is redistributed from slower frequency oscillations to faster frequency oscillations. Slow frequency delta oscillations decreased most with age in the frontal operculum, whereas faster beta band oscillatory power increased most strongly in processing networks and the posterior cingulate cortex. Additionally, we found evidence that developmental decreases in slow frequency coupling between control networks supports the transition from adolescence to adulthood that may be related to age-related improvements in impulse control. Age-related decreases in coupling of these oscillations during the resting state may be a mechanism of increased neural flexibility that occurs during adolescence [57,73,74]. As such, future studies should probe frontal theta as a mechanism by which control instantiation is refined during adolescence, using tasks that probe cognitive flexibility, such as task switching and rapid instructed task learning paradigms [77].

## Methods

### Ethics statement

All subjects gave written informed consent; parent or guardian consent was obtained for all subjects aged 14 to 17 years. The University of Pittsburgh Institutional Review Board (IRB protocol number: PRO10090478) approved the study, adhering to the Declaration of Helsinki. Subjects were compensated monetarily for their participation in the study.

### Subjects

Of the 81 adolescents and adults we recruited for this study, we include data from 68 subjects, ranging in age from 14 to 31 years (M = 22.51, SD = 5.55). Thirteen subjects were dropped due to unavailable ECG and/or electrooculogram (EOG) data. Based on a questionnaire, none of the subjects—nor their first-degree relatives—currently or previously had a psychiatric or neurological disorder.

### Structural MRI acquisition

For each subject, we acquired a structural MRI to coregister MEG data for analyses in source space. Data from the 68 remaining subjects were pooled from 2 separate protocols within the lab and thus had slightly different structural MR sequences, which would not affect subsequent processing steps. For 28 subjects, structural images were acquired using a sagittal magnetization-prepared rapid gradient-echo sequence (repetition time [TR] = 2,100 ms, echo time [TE] = 3.43 ms, flip angle = 8°, inversion time [TI] = 1,050 ms, voxel size = 1 mm isotropic). For the other 40 subjects included in the second protocol, structural images were acquired using a sagittal magnetization-prepared rapid gradient-echo sequence (TR = 2,200 ms, TE = 3.58 ms, flip angle = 9°, TI = 1,000 ms, voxel size = 1 mm isotropic).

### MEG data acquisition

Resting-state MEG data (300 seconds) were acquired using an Elekta Neuromag Vectorview MEG system (Elekta Oy) comprising 306 sensors arranged in triplets of 2 orthogonal planar gradiometers and 1 magnetometer, distributed to 102 locations. The MEG scanner was located inside a 3-layer magnetically shielded room within the University of Pittsburgh Medical Center. The data were acquired continuously with a sampling rate of 1,000 Hz. Head position relative to the MEG sensors was measured continuously throughout the recording period to allow off-line head movement correction. Two bipolar electrode pairs were used to record vertical and horizontal EOG signals to monitor eye movement. A potential confound of developmental studies using MEG is that head size is smaller in younger subjects. Given the sensor locations in the MEG helmet are fixed, smaller heads will by definition have lower signal to noise, as they are further from the sensors. On average, head size is fully developed by 10 years of age [78], which is well below the age of our youngest subject (14 years). We regressed age onto intracranial volume (ICV) and did not observe a significant relationship between ICV and age (*t* = −1.05, *p* = 0.29). Additionally, we regressed ICV onto global theta band (5–9 Hz) PLV and did not observe a significant relationship between ICV and global theta band PLV (*t* = −0.02 *p* = 0.96).

### MEG data processing

For artifact removal, we first manually visually inspected every channel across the resting state run for noisy or flat channels and squid jumps. MEG data were then preprocessed off-line using the temporal signal space separation (tSSS) method (10 second correlation window, 0.98 correlation limit), which uses spatial and temporal features to separate signals into components generated within the MEG helmet and components from outside the helmet, which must be artifactual [79,80]. This method greatly increases the SNR of the resulting data [81]. tSSS also performs head movement compensation by aligning sensor-level data to a common reference [82]. This head motion correction procedure also provides estimates of head motion relative to sensor coordinates that we subsequently used for head motion estimates for each subject. Lastly, the raw time series data were down-sampled to from 1,000 Hz to 250 Hz.

An independent components analysis (ICA) approach was used to automatically detect and attenuate heartbeat, eye blink, and eye movement artifacts. ICA was performed on each channel using the Infomax algorithm, with the number of components selected to account for 95% of the variance. The Pearson correlation of the components and the ECG or EOG channel is used to identify artifactual sources through an iterative thresholding method (as implemented in minimum-norm estimate [MNE] Python [83]) and subsequently manually inspected. After removal of the artifactual sources, the data were reconstructed from the remaining components.

MEG sensor data were then projected from the sensors on to the cortical surface to estimate source activities, using the MNE procedure. First, the geometry of each participant's cortical surface was reconstructed from the respective structural MRI using FreeSurfer [84,85]. The solution space for the source estimation was then constrained to the gray/white matter boundary by placing 5,124 dipoles per hemisphere. A forward solution for the constructed source space was calculated using a single compartment boundary-element model. The noise covariance matrix was calculated from a 2-minute empty room scan, in which we acquired data with no subject present. The noise covariance matrix and the forward solution were then combined to create a linear inverse operator to project the resting-state MEG sensor data to the cortical surface. We then warped individual subject data from native space to FreeSurfer average space to facilitate between-subject interpretation of specific regions and networks.

### ROIs

We extracted the time series of resting-state MEG data from a recent parcellation of 333 ROIs covering the entire cortical surface [45]. This atlas was chosen because it comprises major cortical functional networks, including control networks, processing networks, and the DM network and covers the entire cortical surface. Developmental changes in these networks have been observed in fMRI studies [6,7] and are thus candidates for electrophysiological developmental changes at the timescales of which MEG is sensitive.

### Phase-locking calculation

For each pair-wise relation between ROIs for each subject, a PLV was calculated for each frequency of interest (1–49 Hz in 1-Hz intervals). Phase-locking is a measure of the propensity for 2 signals to maintain a constant phase separation with each other (i.e., synchrony). Therefore, the PLV provides a measure of temporal variability between 2 MEG signals [40].

Here, we binned the data into 100 three-second chunks and obtained 1 PLV across the time windows using a multitapers method with digital prolate spheroid sequence (DPSS) windows (3 tapers), as implemented in MNE python (mne.spectral.connectivity). Three seconds is a sufficiently long segment of data to obtain a reliable estimate of oscillations as low as 1 Hz, as a common recommendation for the minimum number of cycles per window to achieve reliable frequency estimates is 3 [86]. To calculate the PLV at each frequency, 2 time series are spectrally decomposed at a given frequency, given by the equation

$$PLV=\frac{1}{N}\left|\sum_{n=1}^{N}e^{i(\theta 1(n)-\theta 2(n))}\right|$$

where *N* is the number of sampled time points and θ1 and θ2 are the phase values at time point n. The PLV was calculated for each ROI pair, resulting in 55,278 PLVs for each frequency and for each subject. A single averaged PLV was then computed by averaging all of the PLVs, ranging from 0 to 1, representing a random phase relationship and fixed phase relationship, respectively.

### Power calculation

For each ROI, power was calculated using the Welch method (pwelch function in MATLAB) on the 100 three-second chunks of data, with an overlap of 50%. The relative power at each frequency interval in the range of 1–49 Hz (1 Hz bins) was calculated by dividing the power at a given frequency by the total power (summed power) in the 1–49 Hz interval. This value represents the relative magnitude of each frequency in relation to the total signal.

### Determining age-related changes in phase-locking

After ROI × ROI PLV individual subject matrices were calculated at each frequency, individual subject matrices were concatenated forming a 333 × 333 × 49 × 68 four-dimensional matrix. First, we asked whether there were developmental changes in PLV across a broadband frequency range (1–49 Hz). To this end, we averaged the four-dimensional matrix along the first 2 dimensions of the upper triangle, resulting in a single PLV value at each frequency for each subject. A linear mixed-effects model with maximum likelihood estimation was used to examine main effects and interactions predicting PLV. Age and frequency were entered as fixed effects, and random intercepts were estimated for each subject. Significance values for fixed effects were obtained through a likelihood ratio test between models with and without the effects in question (chi-squared test). To test individual frequencies for PLV × Age effects, we regressed PLV against age within each frequency bin and corrected for multiple comparisons using FDR [87]. For visualization purposes in Fig 2A, we performed a median split by age.

### Determining age-related changes in power

First, we asked whether global (across all ROIs) relative power at any frequency interval demonstrated a significant age effect. After relative power was determined for each ROI at each frequency interval, we averaged power across all ROIs for each subject. We then performed a linear regression analysis at each frequency interval (1–49 Hz; 1-Hz bins) and corrected for multiple comparisons using an FDR correction [87]. For visualization purposes in Fig 2B, we performed a mediation split by age.

### Anterior-to-posterior gradient of decoupling across development

Once we determined the frequency ranges of significant age effects in phase-locking (theta band: 5–9 Hz) and power (delta band: 1–3 Hz; beta band 14–16 and 22–26 Hz), we sought to determine the specific regions in which phase-locking and power were significantly changing with age. For the analysis of power, for each ROI, we ran linear regression models to determine the rate of change in power within each frequency band as a function of age and extracted the beta weight value from the age regressor. This resulted in a beta weight matrix (ROI × Frequency). We then summed across frequencies within the range of significant effects (e.g., 1–3 Hz for delta band power) for each ROI. For the phase-locking analysis, we ran linear regression models to determine the rate of change in PLV within the theta band as a function of age, controlling for potential confounds, including motion, power, and distance (see below). This resulted in a 333 × 333 matrix of beta weights from the age regressor, representing the rate of change in phase-locking for every ROI pair. To obtain a summary statistic for each ROI, we summed down each column of the matrix, resulting in 333 summed beta weights, which we use to characterize the summed rate of change with age for every ROI across the cortical surface. This process was repeated across frequencies of interest (1–49 Hz) by averaging across frequencies in 5 Hz bins (i.e., 1–5 Hz, 6–10 Hz, …, 46–49 Hz).

We were interested in general trends across the cortical surface. To this extent, we calculated the center of mass for every ROI to obtain a center coordinate and to also get a measure of Euclidean distance between each ROI pair. We the regressed the y-coordinate of the ROI onto the summed beta weights for each ROI (for power and phase analyses separately), controlling for average distance between ROIs and ROI surface area. The average distance between ROIs was included as a nuisance regressor to attenuate the effects of volume conduction. For the PLV analysis, this process was also repeated at each frequency interval and across 5 Hz frequencies bins in the range of 1–49 Hz to determine the specificity of the anterior-to-posterior gradient to the theta band. Specifically, we tested for a significant difference between the slope of each regression model (i.e., beta weights) versus the model including the theta band (6–10 Hz for this analysis) using the following formula [88]:

$$z=\frac{\beta_{1}-\beta_{2}}{\sqrt{{SE\beta }_{1}^{2}+{SE\beta }_{2}^{2}}}$$

where z is equal to the test statistic (values > 1.645 correspond to *p* < 0.05, one-tailed), β_1_ is equal to the regression coefficient of the y-coordinate in the 6–10 Hz interval, β_2_ is equal to the regression coefficient of the y-coordinate in the test interval (e.g., 1–5 Hz), SEβ_1_^2^ is the squared standard error of the β_1_ coefficient, and SEβ_2_^2^ is the squared standard error of the β_2_ coefficient.

### Specific ROI interactions driving regional changes in PLV

Next, we wanted to identify any trends in specific ROI pairs driving regional decreases in phase-locking. First, we sorted ROIs according to the magnitude of the summed beta weights. We then further probed the top 5% of these ROIs (*n* = 16), which represents the 16 ROIs undergoing the greatest amount of developmental decrease in phase-locking. Of those 16 ROIs, we further thresholded each ROI’s specific interactions with other ROIs to maintain only the top 5% of each ROIs pairwise beta weight (*n* = 16 pairwise interactions for each of the 16 ROIs), resulting in a total of 256 pairwise beta weights demonstrating the greatest rate of ROI-ROI decrease in phase-locking.

### Control for power in PLV × Age models

We wanted to ensure any age-related changes we observed in PLV was not due to changes in the total amount of activity (power) in an area within any given frequency band [64]. First, we extracted a power estimate for each ROI. Specifically, we calculated relative power (see “Power calculation”). We then extracted relative power in the 5–9 Hz frequency band within subjects by taking the mean power within this frequency range for each ROI and dividing by broadband total power (1–49 Hz) for each ROI. For each ROI within each subject, this procedure resulted in relative theta band power. We then averaged across subjects to obtain a mean relative theta band power for each ROI. This value was then plotted against each ROIs y-coordinate to determine the anterior-to-posterior gradient in power across the cortex. Because a significant anterior-to-posterior gradient in power was observed (more power in posterior regions), we included as nuisance regressors the power of each ROI, the interaction between each ROI pair, the log-transformed power of each ROI, and the log-transformed interaction term of each ROI pair into the age models for each ROI pair. Additionally, matching the PLV analysis pipeline, we regressed power onto age at every frequency interval ranging from 1–49 Hz in 1 Hz increments.

### Head movement correction

During MaxFilter preprocessing, continuous head position estimates are calculated, and any large or sudden head movements are recorded. While MaxFilter performs head movement correction by aligning sensor data to a common reference, it does not account for artifacts generated by head movements, and we wanted to ensure any effects were not a result of head motion artifacts. After extracting the estimated movements from the MaxFilter output, we used the translation vector and rotation matrix for the head position relative to the sensor array (obtained from coregistration) to calculate a three-dimensional head movement vector relative to each sensor at each time point. The norm of this movement vector was averaged across sensors to obtain a single measure of head motion. This motion estimate for each subject was included as a nuisance regressor in all regression models involving the analysis of age-related changes in PLV.

### Relationship of impulsivity with PLV and power

Prior to the neuroimaging visit (M = 43.61 days, SD = 43.33 days), a subsample of participants (*n* = 62) completed the UPPS-P Impulsive Behavior Scale [48,89–92], either in an online screening (*n* = 28) or a separate behavioral visit (*n* = 34). In the current analysis, total impulsivity scores were estimated according to procedures outlined by [48]. We then regressed age onto this total impulsivity score and observed a significant negative linear relationship between total impulsivity and age (see Results).

To determine overlap between links demonstrating a significant PLV × Age relationship and a significant PLV × Impulsivity relationship in a nonarbitrary, data-driven manner, individual subject theta band PLV matrices were submitted to the NBS [50], and a *t* test was run between adolescents and adults to extract a cluster of regions with a significant decrease in theta PLV with age. We then performed the NBS on the relationship between impulsivity and theta PLV, controlling for age. A total of 3 connections overlapped between the 2 models and were subsequently confirmed using mediation analysis.

To examine whether differences in PLV may account for age-related differences in impulsivity, mediation analysis was performed on PLV values within connections that had significant associations with (1) age and (2) impulsivity (while controlling for age), as defined above. Significance values for indirect effects were obtained using 5,000 draws in a bootstrap procedure [93].

To determine whether resting-state delta band or beta band power mediated the relationship between age and impulsivity, similar to the PLV analysis, we tested each ROI across these 2 frequency bands for mediation effects. Significance values for indirect effects were obtained using 5,000 draws in a bootstrap procedure, as was done previously.

### Working memory task

The spatial working memory task was modeled on the classic Sternberg working memory paradigm. Cue stimuli were yellow circles appearing in 1 of 8 possible locations. Each trial began with fixation followed by a presentation of 3 frames (300 ms each) showing one cue stimulus at a time in either the same location or 3 different locations. A blank grid was inserted between the frames for 200 ms to decrease chunking and motion perception. A 1,500 ms (50% of trials), 3,000 ms (25% of trials), or 4,500 ms (25% of trials) delay period was used to minimize habituated preparatory responses.

Following the delay period, subjects made a button press to indicate whether a frame showing 4 circles located among 8 possible locations had occurred in any of the previous cue locations (50% of trials) or were all in novel locations (50% of trials). A total of 144 high load trials and 144 low load trials were distributed across 12 runs, with the order randomized within runs. Intertrial fixation intervals ranged between 1,000 and 4,500 ms, with a short break between runs. The task was designed and run using E-Prime (Psychology Software Tools, Inc., Pittsburgh, PA).

### Task MEG data preprocessing

MEG data were first manually inspected for flat or noisy channels that can arise due to sensor malfunction, and these channels were removed from further analysis, as excessively noisy or flat channels may adversely impact further preprocessing steps and data analysis. The maximum number of channels excluded within a single participant was 23. As we did with the resting-state data, we attenuated environmental noise using the MaxFilter software to apply tSSS [80]. If at any time during a trial the total displacement of MEG sensors relative to the head was greater than 5 mm, the trial was rejected from all future analyses. Across all participants, only 38 total trials were dropped for head motion, with at most 4 trials dropped for head motion within a single participant.

The remaining preprocessing steps were applied using tools in the MNE Python package [83]. First, the data were band-pass filtered to the frequency range of interest (1–49 Hz) using a 10-second overlap-add FIR filter. Cardiac, eye blinks, and eye movement (saccade) artifacts are not identified by tSSS because they originate from the subject's body, so we used an ICA method to attenuate these artifacts, similar to the resting-state methods. The shapes of the automatically detected artifactual components were checked visually to verify the selection of artifactual components, and the selection of components was then amended in the rare cases that the automatic procedure failed to identify components that showed clear EOG or ECG patterns. Finally, trials were screened for remaining sensor jumps, muscle artifacts, or saccade artifacts by checking for magnetometer amplitudes that exceeded 2.5 × 10^−10^ T or gradiometer amplitudes that exceeded 4 × 10^−10^ T/m; no further trials were rejected by these criteria.

During the experiment, trial event onset times were recorded into a digital stimulus channel through the E-Prime software. The event timings and codes from this channel were checked against E-Prime log files to remove spurious events that occurred in some runs due to software timing synchronization glitches. Based on this verified trial event data, trials with incorrect or omitted responses were removed because we are interested only in trials during which working memory was successfully engaged. In addition, a total of 10 trials across all participants were rejected due to mismatches between stimulus channel event codes and timing reported by E-Prime, with at most 4 trials dropped from a single subject for this reason.

After preprocessing, we extracted the first 1,500 ms of the maintenance period from the task and calculated the PLV between each of the 333 ROIs in the 5–9 Hz frequency range, following the resting-state analysis pipeline. For each ROI pair, we then regressed the PLV onto age, controlling for subject head motion. Next, the beta weight from the age regressor was extracted from each model, and beta weight matrices were constructed. As in the resting-state analysis, we summed down the columns of the matrix to get a summed beta weight representing the total linear age effect. We then regressed this value for ROI against the ROI’s anatomical y-coordinate and did not observe any anterior-to-posterior effects (*t* = −0.02, *p* = 0.98).

## Supporting information

S1 Data Data points for figures. (XLSX)

## Abbreviations

ACC: anterior cingulate cortex
aIns: anterior insula
BOLD: blood oxygen level–dependent
CP: Cinguloparietal
DM: default mode
DPSS: digital prolate spheroid sequence
EEG: electroencephalography
EOG: electrooculogram
FDR: false discovery rate
fMRI: functional magnetic resonance imaging
FP: Frontoparietal
ICA: independent components analysis
ICV: intracranial volume
MEG: magnetoencephalography
MNE: minimum-norm estimate
MNI: Montreal Neurological Institute
PLV: phase-locking value
ROI: region of interest
rs-fMRI: resting-state fMRI
RT: Retrospenial temporal
SAL: salience
SMH: Somatomotor Hand
SMM: Somatomotor Mouth
SNR: signal-to-noise ratio
TE: echo time
TI: inversion time
TR: repetition time
tSSS: temporal signal space separation
VA: ventral attention
